# PTP4A3, A Novel Target Gene of HIF-1alpha, Participates in Benzene-Induced Cell Proliferation Inhibition and Apoptosis through PI3K/AKT Pathway

**DOI:** 10.3390/ijerph17030910

**Published:** 2020-02-01

**Authors:** Yunqiu Pu, Fengxia Sun, Rongli Sun, Zhaodi Man, Shuangbin Ji, Kai Xu, Lihong Yin, Juan Zhang, Yuepu Pu

**Affiliations:** Key Laboratory of Environmental Medicine Engineering, Ministry of Education, School of Public Health, Southeast University, Nanjing 210009, China; tina91@126.com (Y.P.); fxsun7@outlook.com (F.S.); sunrongli20609@163.com (R.S.); 13951023277@163.com (Z.M.); JSBIN5708@163.com (S.J.); 230189311@seu.edu.cn (K.X.); lhyin@seu.edu.cn (L.Y.); 101011288@seu.edu.cn (J.Z.)

**Keywords:** Benzene, HIF-1alpha, PTP4A3, Cell proliferation, Apoptosis

## Abstract

Benzene, a commonly used chemical, has been confirmed to specifically affect the hematopoietic system as well as overall human health. PTP4A3 is overexpressed in leukemia cells and is related to cell proliferation. We previously found that HIF-1alpha was involved in benzene toxicity and PTP4A3 may be the target gene of HIF-1alpha via ChIP-seq. The aim of this study is to confirm the relationship between HIF-1alpha and PTP4A3 in benzene toxicity, as well as the function of PTP4A3 on cell toxicity induced by 1,4-benzoquinone (1,4-BQ). Our results indicate that HIF-1alpha could regulate PTP4A3 with in vivo and in vitro experiments. A cell line with suppressed PTP4A3 was established to investigate the function of PTP4A3 in 1,4-BQ toxicity in vitro. The results revealed that cell proliferation inhibition was more aggravated in PTP4A3 low-expression cells than in the control cells after 1,4-BQ treatment. The relative oxygen species (ROS) significantly increased in cells with inhibited PTP4A3, while the rise was inferior to the control cells at the 20 μM 1,4-BQ group. An increase in DNA damage was seen in PTP4A3 down-regulated cells at the 10 μM 1,4-BQ group, whereas the results reversed at the concentration of 20 μM. Moreover, the apoptosis rate increased higher in down-regulated PTP4A3 cells after 1,4-BQ exposure. In addition, PI3K/AKT pathway was significantly restrained in cells with inhibited PTP4A3 after 1,4-BQ treatment. Our results indicate that HIF-1alpha may regulate PTP4A3 to be involved in benzene toxicity. Inhibition of PTP4A3 could aggravate cell proliferation suppression and apoptosis by regulating PI3K/AKT pathway after 1,4-BQ treatment.

## 1. Introduction

Benzene is widespread in industry and living environments. Long-term exposure to benzene results in hematologic damage, which is mainly displayed by the decrease in all three kinds of blood cell in circulating blood. Reduction in the three cell types is regarded as the result of aplastic anemia, myelodysplastic syndromes or acute myelocytic leukemia (AML) [1,2,3]. In China, millions of workers are exposed to benzene in their workspace. However, the exact mechanism of benzene toxicity is unknown. It is important to explore the mechanism, so effective measures could be carried out to protect workers exposed to benzene.

Bone marrow is determined to be the target organ for benzene toxicity [4]. The metabolism of benzene begins in the liver and yields benzene oxide, which can then transformed to phenol. Phenol can be hydroxylated to form hydroquinone, which can then be oxidized to 1,4-benzoquinone (1,4-BQ) [5,6]. It is generally accepted that the myelotoxic effect of benzene is mainly caused by 1,4-BQ [7]. Amounts of reactive oxygen species (ROS) are generated during benzene metabolism. ROS has been confirmed to be an important factor involved in benzene-induced leukemia [8].

HIF-1alpha is activated in a hypoxic environment as a transcription factor and participates in several programs to ensure hypoxic cell survival [9,10,11]. The hypoxic response is thought to protect hematopoiesis stem cells from oxidative stress [12,13]. ROS increase was seen in hematopoietic stem cells (HSC) lack of HIF-1alpha and resulted to the HSC silence reduction and apoptosis increase [14]. In our previous study, it was found that HIF-1alpha may participate in benzene-induced mouse hematopoietic inhibition with increase oxidative stress [15,16]. 

PTP4A3 has been reported to have a positive relationship with HIF-1alpha in gastric cancer through gene set enrichment analysis (GSEA) [17]. Protein tyrosine phosphatase of regenerating liver 3 (PRL-3, encoded by PTP4A3 gene) is a member of the VH1-like protein tyrosine phosphatase (PTP) family with dual specificity [18]. Studies have reported that PTP4A3 is frequently overexpressed in numerous tumor samples, such as the intestine, stomach, lung, bladder, breast, ovary and liver [19,20]. Several studies have also shown that PTP4A3 participates in the development of acute myeloid leukemia and chronic myeloid leukemia [21,22,23]. PTP4A3 is also thought to be associated with cell proliferation and apoptosis. Overexpression of PTP4A3 was found in half of AML bone marrow samples, which have the characteristics of evasion of apoptosis and unlimited proliferation [24,25]. The suppression of PTP4A3 induced growth inhibition of the prostate cancer cell lines in vitro [26,27]. Other studies showed that overexpression of PTP4A3 promoted cancer cell proliferation [28,29]. PI3Ks are a family of enzymes related to several cellular functions, including cell proliferation and apoptosis. AKT is known as a kinase which maintains the balance between cell survival and apoptosis. PTP4A3 was reported to activate the PI3K/AKT pathway in colorectal cancer cells [30].

Benzene is a common chemical compound that exists in the environment. It is important to investigate the precise mechanism of benzene toxicity in order to prevent benzene-induced hematopoietic damage. Many potential mechanisms have been considered to be involved in benzene toxicity [6,31,32], but the exact mechanism remains unknown. PTP4A3 was not reported to participate in benzene toxicology in the past. In this paper, we further verified that PTP4A3 may participate in benzene toxicity by the regulation of HIF1-alpha through in vivo and in vitro experiments. We established the benzene-poisoning mice model and a K562 cell line with inhibited PTP4A3 to investigate the function and mechanism of PTP4A3 in benzene toxicity. In addition, PTP4A3 influenced benzene-induced proliferation inhibition and abnormal apoptosis via PI3K/AKT pathway. In this article, the effect and mechanism of PTP4A3 in benzene toxicity were preliminarily investigated. It is helpful to investigate the precise mechanism of benzene toxicity.

## 2. Materials and Methods

### 2.1. Establishment of Benzene Poisoning Mice Model

Male C57BL/6 mice were obtained from the Laboratory Animal Center of Nanjing University. The benzene poisoning mice model was established as previously described [33]. There was random allocation into two groups: control (vehicle: oil) and benzene-exposure (150 mg/kg). The mice were subcutaneously injected with benzene dissolved in corn oil daily for 15 consecutive days. The weight of mice was measured every five days. After 15 days, mice blood was collected for hematological analysis. Routine blood examination was detected by an automated blood analyzer. The mice were sacrificed and the main organs, as well as the thymus, were excised and weighed. Bone marrow cells were collected for subsequent studies. 

### 2.2. Cell Culture 

K562 cell line was used in vitro for the experiment. The K562 cell line was obtained from the American type culture collection (ATCC). The cells were cultured in IMDM medium. The culture medium contained 10% fetal bovine serum and 1% penicillin/streptomycin. The cells were cultured in the environment with 5% CO_2_ at 37 °C.

### 2.3. Lentiviral Transduction for PTP4A3 Knockdown

**Figure 1:**
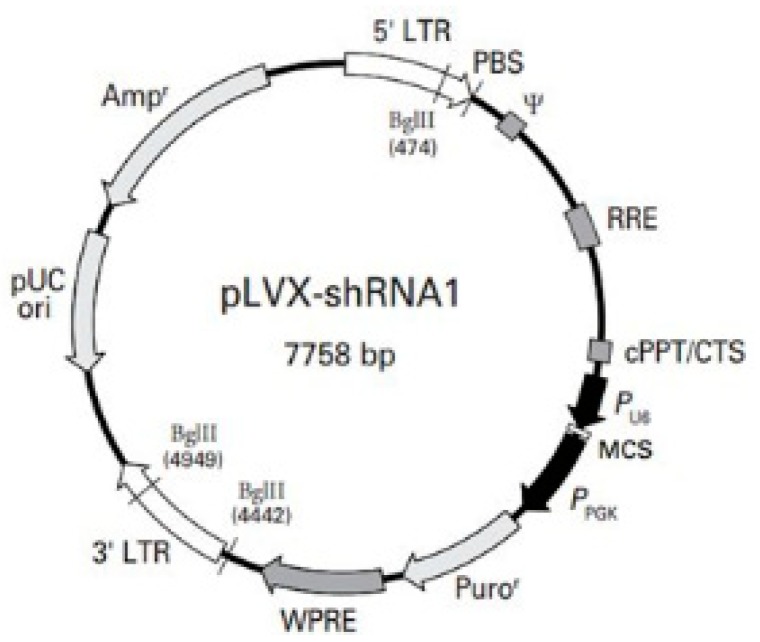
The vector map of shRNA designed to knockdown PTP4A3.

The lentiviral was designed and packaged by Genecreate (Wuhan, China). The vector map of shRNA is shown in Figure 1. The sequence of shRNA is as follows: gctacaaacacatgcgcttcc. K562 cells were transduced with viruses produced by packaging cells in order to establish the K562 cell line with down-regulated PTP4A3 (shRNA PTP4A3) and a control cell line (pLKO K562).

### 2.4. Cell Proliferation Assay

The cells were adjusted to 5 × 10^5^/mL and seeded in 96-well microplates. Different concentrations of 1,4-BQ (0, 10, 20, 40, and 80 μM) were added into the cell culture medium and kept for 24 h. Cell viability was measured using MTT. The 20 μL MTT (5 mg/mL) solution was added to each well. After homogeneous mixing, the cells were cultured at 37 °C for another 4 h. The cells were mixed with 150 μL DMSO to dissolve the sediment and then determined the absorbance at a 490nm wavelength.

### 2.5. 5-Ethynyl-2’-deoxyuridine(EdU) Assay

EdU assay was performed through EdU DNA Proliferation in vitro Detection (RiboBio, Guangzhou, China) based on the manufacturer’s instructions. In brief, the cells were fixed with 4% paraformaldehyde after the incubation with 50 μM EdU for 2 h. The fixed cells were neutralized with 2 mg/mL glycine for 5 min, and then treated with 0.5% Triton X-100 for another 10 min. Next, the cells were stained with Apollo^®^ dye in the dark for 10 min at room temperature and washed with 0.5% Triton X-100 three times. The labeled cells were then analyzed by flow cytometry.

### 2.6. Apoptosis

The apoptosis of cells was tested using Annexin V Apoptosis Detection Kit (BD Biosciences, San Jose, CA, USA). The procedure follows the manufacturer’s instructions. The cells were collected and washed with pre-chilled PBS twice, and then adjusted to the concentration of 1 × 10^6^ cells/mL with Binding Buffer. FITC Annexin V and PI were used to identify the cell apoptosis and cell death. The cells were incubated in the dark for 15 min at room temperature and then 400µL of Binding Buffer was added. The apoptosis of labeled cells was analyzed by flow cytometry within one hour (BD Biosciences, San Jose, CA, USA).

### 2.7. Real-Time Quantitative PCR (qPCR) Detecting System

The RNA was extracted by TRIzol^®^ Reagent (Life Technologies, Carlsbad, CA, USA) and reverse transcribed to cDNA using Takara cDNA Reverse Transcription Kit (Takara Bio, Shiga, Japan) according to the manufacturer’s instructions. The qPCR was performed based on the resulting cDNA with specific primers in the Step One Plus Real-Time PCR System (Applied Biosystems, Foster City, CA, USA). Predesigned primer pairs for mouse and human PTP4A3, mouse and human β-Actin, were purchased from Sangon (Shanghai, China). The primers for each gene are listed in Table 1. According to the manufacturer’s instructions, each well required 20 μL reaction mixture, including the following components: 10µL of SYBR Premix Ex Taq II, 0.8µL of each forward and reverse primer, 0.4µL ROX Dye and 500 ng template cDNA. The qPCR procedure was designed as below: the denaturation stage was 95 °C for 5 s, followed by the amplification stage (95 °C for 15 s, 60 °C for 60 s and 95 °C for 15 s) with 40 cycles. The 2^−ΔΔCt^ method was used to analyze the gene expression. The expressions of target genes were normalized based on β-Actin. The results were adjusted according to the primer efficiencies previously calculated.

### 2.8. Protein Extraction and Western Blotting

Proteins were isolated from cells and mice using the Radio Immunoprecipitation Assay (RIPA). The protein concentration was determined by BCA assay, and then adjusted to the same concentration. The last step of sample preparation was boiling the protein with loading buffer for 5 min.

Different proteins were separated by electrophoresis on 10% SDS-PAGE gels and transferred to polyvinylidene difluoride (PVDF) membranes at a constant voltage. Blots were blocked in Tris-Buffered Saline Tween-20 (TBST) with 5% non-fat milk and further incubated with the primary antibodies overnight at 4 °C. After washing with cold TBST, the membranes were incubated with horseradish peroxidase (HRP)-conjugated secondary antibody for one hour at room temperature. The fluorescence was detected with electrochemiluminescence (ECL) fluid. The internal control was glyceraldehyde-3-phosphate dehydrogenase (GAPDH) to confirm that each sample contained the same concentration of protein. Antibodies against PI3K, AKT, p-AKT, and PTP4A3 were obtained from Cell Signaling Technology (Danvers, MA, USA). The antibody against GAPDH was from Abcam (Cambridge, MA, USA). The antibody against HIF-1alpha was from Novus Biological (Littleton, CO, USA).

### 2.9. Chromatin Immunoprecipitation and Quantitative PCR (ChIP-qPCR) 

Chromatin immunoprecipitation (ChIP) was conducted by CloudSeq Biotech Inc. (Shanghai, China). In brief, the procedure contained six steps. First, mice bone marrow cells were cross-linked with 1% formaldehyde for 10 min, and then glycine was added to termination. Protease inhibitor cocktail was added into the sample after washing with PBS. Nuclei were subjected to ultrasonication after isolation and digestion. The sonicated chromatin was purified with a mixture of phenol, chloroform and isopropanol, and then the concentration was determined with Nanodrop. The specific antibody was added into the prepared sample and mixed together at 4 °C in a rotator overnight. After incubation, the samples were eluted and purified according to the manufacturer’s instructions. Finally, the purified DNA was analyzed by qPCR. The routine of qPCR was the same as described above.

### 2.10. Comet Assay

Comet assay was performed using an OxiSelect™ Comet Assay Kit (Cell Biolabs, San Diego, CA, USA). The operation followed the manufacturer’s instructions. In brief, cells were resuspended in cold PBS at a concentration of 1 × 10^5^ cells/mL. Comet Agarose was preheated and then combined with cell samples at 1:10 ratio (v/v). The mixture immediately transferred onto the top of the Comet Agarose Base Layer in avoidance of solidification. The slide transferred to 4 ℃ in the dark for 15 min. The slide was maintained in a container loaded with pre-chilled Lysis Buffer for another hour at 4 °C in the dark. Alkaline electrophoresis was performed after the slide was kept in the container with pre-chilled Alkaline Solution at 4 °C for 30 min in the dark. Each well was stained with 100 μL diluted Vista Green DNA Dye at room temperature for 15 min. The slide could be observed by epifluorescence microscopy using a FITC filter. At least 50–100 cells were analyzed per sample.

### 2.11. ROS Detection

ROS detection was performed using a ROS detection kit (KeyGEN BioTECH, Jiangsu, China). The procedure followed the manufacturer’s instructions. The cells were suspended and seeded in 6-well microplates at the appropriate concentration. Different concentrations (10 μM, 20 μM) of 1,4-BQ were used to treat the cells for 2 h. Cells were resuspended in 10 μM DCFH-DA and incubated in the dark at 37 °C for 20 min as well as blended every three minutes. The labeled cells were analyzed by flow cytometry.

### 2.12. Statistics

The results were analyzed using SPSS 19.0 (IBM Corp, Chicago, IL, USA). All data were presented as the mean±SD. The statistical analysis was performed through one-way ANOVA or non-paired T-test. A level of significance of *p* < 0.05 was used for all analyses. 

## 3. Results

### 3.1. Mice Model of Benzene Poisoning

**Figure 2:**
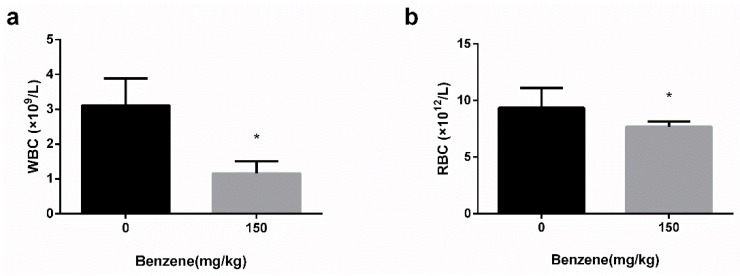
White blood cells (WBC) and red blood cells (RBC) reduced in mice after benzene exposure. (**a**) WBC and (**b**) RBC of mice were measured after daily subcutaneous injection of benzene or corn oil for 15 days. *: *p* < 0.05, compared with control group.

To establish the benzene-poisoning model, on a daily basis for half a month, mice were treated with corn oil dissolved with benzene via subcutaneous injection. After 10 days, the mice with benzene exposure showed a slower weight increase than the control group. The weight of benzene exposed mice was generally lighter than the control group after 15 days (Data not shown). A significant decrease in white blood cells (WBC) and red blood cells (RBC) was observed in benzene-exposed mice after 15 days (Figure 2).

### 3.2. PTP4A3 Participated in Benzene Toxicity via HIF-1alpha Regulation

**Figure 3:**
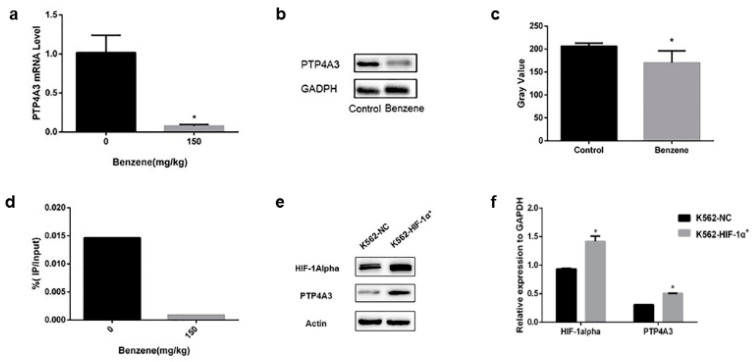
PTP4A3 participated in benzene toxicity under the regulation of HIF-1alpha. (**a**) The mRNA level of PTP4A3 was measured with real-time quantitative PCR. (**b**,**c**) The expression of PTP4A3 in benzene-exposed mice was measured with western blot. (**d**) The relationship between HIF-1alpha and PTP4A3 in benzene exposed mouse bone marrow was measured with ChIP-qPCR. (**e**,**f**) The expression of PTP4A3 was measured in HIF-1alpha high expression K562 cells (K562-HIF-1α^+^). The β-actin was applied to internal reference. *: *p* < 0.05, compared with the control group.

To determine whether HIF-1alpha regulated PTP4A3 to be involved in benzene toxicity, we measured the expression of PTP4A3 both in vivo and in vitro. At first, we measured the expression of PTP4A3 and HIF-1alpha in bone marrow cells from mice exposed to benzene. The mRNA and protein level of PTP4A3 declined significantly in mouse bone marrow cells in the benzene-exposed groups (Figure 3a–c). We previously found that HIF-1alpha expression was reduced in benzene-exposed mice [15]. These results indicate that benzene exposure suppressed HIF-1alpha and PTP4A3 expression in bone marrow cells. We found that PTP4A3 was one of the target genes of HIF-1alpha via ChIP-seq [34]. To further confirm the relationship between HIF-1alpha and PTP4A3, we used bone marrow cells separated from benzene toxic mice, or negative control mice, to administrate the ChIP-qPCR. ChIP-qPCR can effectively verify the binding level of HIF-1alpha and its response gene. The results indicate that the fold of PTP4A3 enriched by HIF-1alpha antibody in mouse bone marrow cells in the benzene exposure group was 0.03 times of that in the control group (Figure 3d). 

To further verify the relationship between HIF-1alpha and PTP4A3 in vitro, we tested the expression of PTP4A3 in previous established HIF-1alpha overexpression K562 cells. A significant increase in PTP4A3 expression was observed in HIF-1alpha up-regulated cells (Figure 3e,f).

### 3.3. Establishment of Down-Regulated PTP4A3 Cell Line 

**Figure 4:**
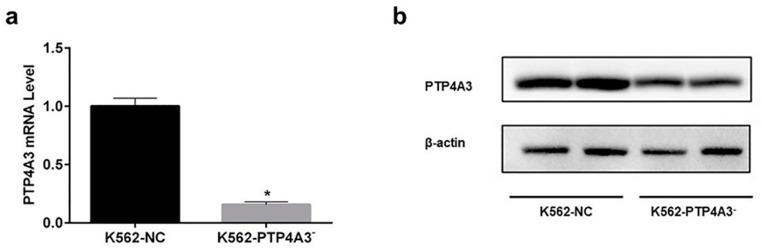
Establishment of PTP4A3 down-expression K562 cell line. (**a**) The expression of PTP4A3 at mRNA level was measured with real-time quantitative PCR. (**b**) The expression of PTP4A3 was measured with western blot. *: *p* < 0.05, compared with the K562-NC cells.

To explore the effect of PTP4A3 in 1,4-BQ cell toxicity in vitro, we established the PTP4A3 down-regulated cell line (K562-PTP4A3^−^) through lentivirus vector transfection. ‘K562-NC’ represents the cells which were transfected with the empty lentivirus vector. The expression of PTP4A3 was significantly reduced in both mRNA and protein (Figure 4). The results indicate that the PTP4A3 down-regulation cell model was successfully established.

### 3.4. Down-Regulation of PTP4A3 Aggravated 1,4-BQ-induced Reduction in Cell Viability and Proliferation

**Figure 5:**
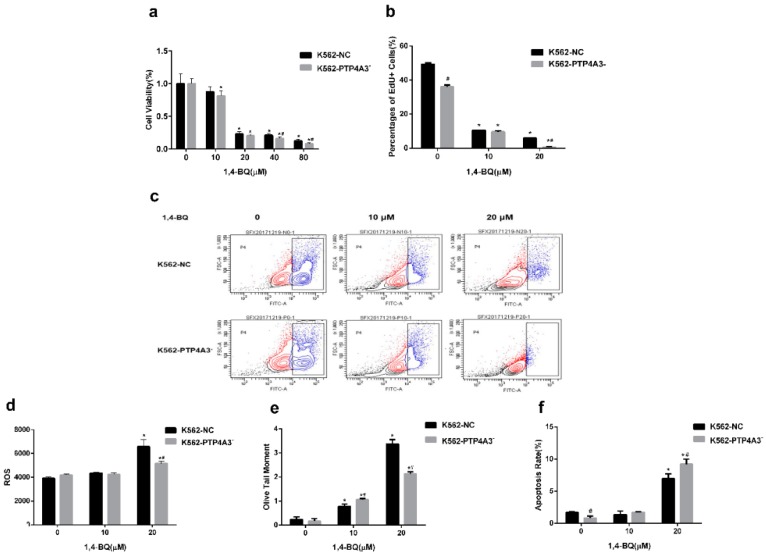
Effects of inhibition of PTP4A3 on the cell proliferation, ROS production, DNA damage and apoptosis after 1,4-BQ treatment. (**a**) The relative cell viability rate was measured by MTT after 1,4-BQ treatment for 24 h. (**b**) The percentage of EdU positive cells was detected by flow cytometer after 1,4-BQ exposure for 24 h. (**c**) The results of EdU recorded from flow cytometry. (**d**) Mean fluorescence intensity of ROS was detected by flow cytometer after 2 h 1,4-BQ treatment. (**e**) DNA damage was tested with comet assay. Olive tail moment was measured to evaluate the DNA damage. (**f**) The percentage of apoptotic cells was tested by flow cytometer after treated with 1.4-BQ for 24 h.*: *p* < 0.05, compared with 0 μM group; #: *p* < 0.05, compared with K562-NC cells at the same concentration of 1,4-BQ.

Since PTP4A3 was related to the cell proliferation, we then investigated the cell growth change after exposure to different concentrations of 1,4-BQ for 24 h. The cell proliferation was obviously restrained by the 1,4-BQ concentration increase. When 1,4-BQ was 40 μM and 80 μM, the relative growth rate of K562-PTP4A3^−^ cells was significantly lower than K562-NC cells (Figure 5a). Based on the cytotoxicity reflected by the MTT assay results, we selected concentrations below 40 μM in subsequent experiments.

EdU is a thymine analogue which can replace the thymine in DNA replication. EdU is commonly used to investigate the cell DNA replication activity. After exposure to 10 μM or 20 μM 1,4-BQ for 24 h, the DNA replication activity of both K562-PTP4A3^−^ cells and K562-NC cells were significantly reduced. At the concentration of 20 μM, the DNA replication activity of K562-PTP4A3^−^ cells was remarkably lower than K562-NC cells (Figure 5b,c).

### 3.5. The effects of Down-Regulation of PTP4A3 on ROS Production, DNA Damage and Apoptosis after 1,4-BQ Exposure

ROS plays an important role in the toxic effect of 1,4-BQ, so we tested the ROS level in K562-PTP4A3^−^ and K562-NC cells after 1,4-BQ exposure. There was no variation in both cell lines at 10 μM 1,4-BQ exposure. ROS level notably increased in both K562-PTP4A3^−^ cells and K562-NC cells after two-hour exposure to 20 μM 1,4-BQ while less ROS increase was seen in K562-PTP4A3^−^ cells compared the control cells. (Figure 5d).

To investigate whether DNA damage is one of the reasons which caused more serious cell proliferation suppression in K562-PTP4A3^−^ cells, a comet assay was used to detect the DNA damage of both cell lines after exposure to 1,4-BQ. An increase in DNA damage was seen in both K562-NC cells and K562-PTP4A3^−^ cells after 1,4-BQ treatment and was related to the dose. The damage was higher in PTP4A3 low-expression cells at the low dose exposure (10 μM), whereas the result was opposite at a high dose (20 μM) (Figure 5e). This shows that 1,4-BQ can cause DNA damage. The DNA damage was exacerbated at low dose with PTP4A3 down regulation.

ROS and DNA damage could cause apoptosis, so this was tested. K562-PTP4A3^−^ cells and K562-NC cells were treated with 10 μM and 20 μM of 1,4-BQ, respectively, for 24 h. PI and FITC fluorescence were tested via flow cytometry to detect the apoptosis rate. The early apoptosis rate was slightly lower in K562-PTP4A3^−^ cells than K562-NC cells in the normal medium culture. The apoptosis rate had no significant difference at a low dose (10 μM) of 1,4-BQ, while it was enhanced at a high dose (20 μM). Compared to K562-NC cells, 1,4-BQ caused more apoptosis in K562-PTP4A3^−^ cells at the concentration of 20 μM. This indicates that a large dose of 1,4-BQ induced apoptosis increase and the situation was worse with the inhibition of PTP4A3 (Figure 5f).

### 3.6. PTP4A3 Participated in 1,4-BQ-induced Cytotoxicity through PI3K/AKT Pathway

We conjectured that the PI3K/AKT pathway was influenced by PTP4A3 inhibition with 1,4-BQ exposure. Therefore, we detect the expression of key proteins in PI3K/AKT pathway by western blot. After 24 h of different concentrations of 1,4-BQ exposure, a significant decrease expression of PTP4A3 was observed in K562-NC cells either, which indicated 1,4-BQ could inhibit PTP4A3. The expression of PI3K was obviously reduced in K562-PTP4A3^−^ cells compared to K562-NC cells after the two types of cell were treated with 10 μM, 20 μM of 1,4BQ. 

**Figure 6:**
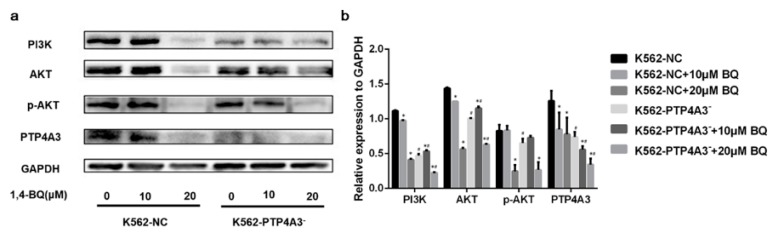
PI3K/AKT pathway was affected by PTP4A3 inhibition after 1,4-BQ exposure. (**a**,**b**) The expression of PTP4A3 and key proteins in PI3K/AKT pathway after 1,4-BQ treatment. GAPDH was applied to internal reference. *: *p* < 0.05, compared with 0 μM group; #: *p* < 0.05, compared with K562-NC cells at the same concentration of 1,4-BQ.

Expression of key proteins in the PI3K/AKT pathway, such as PI3K, total AKT, and phosphorylated AKT were obviously inhibited after 1,4-BQ exposure in both cell lines. It is noteworthy that the PI3K expression in K562-PTP4A3^−^ cells was lower than in K562-NC cells at the same 1,4-BQ level (Figure 6).

## 4. Discussion

Benzene exposure is unavoidable in both occupational and environmental settings and long-term exposure may cause hematopoietic disorder [2,35,36]. In this study, we found that PTP4A3, as a target gene of HIF-1alpha, participated in benzene hematopoiesis damage. In addition, we inhibited PTP4A3 expression in the K562 cell line to investigate the function of PTP4A3 in benzene toxicity. The results show that PTP4A3 could intensify the proliferation suppression and apoptosis caused by 1,4-BQ through prohibition of PI3K/AKT pathway.

HIF-1alpha is vital in retaining the hypoxia environment of bone marrow to survive the hematopoietic stem cell. We previously found HIF-1alpha was distinctly down-regulated in benzene exposed mice, suggesting HIF-1alpha may be involved in benzene toxicity [15,16]. In addition, PTP4A3 was considered to be the target gene of HIF-1alpha through ChIP-seq [34]. Another paper has reported that PTP4A3 and HIF-1alpha are highly related in gastric cancer migration and invasion progress [17]. In this study, we found that PTP4A3 and HIF-1alpha had a positive relationship in vivo and in vitro, which indicated that HIF-1alpha may regulate PTP4A3 to be involved in benzene toxicity.

PTP4A3 was highly expressed in numerous cancer types. A previous study reported ovarian cancer cells with PTP4A3 inhibited resulted in cell proliferation suppression [37]. A similar result was reported in hepatocellular carcinoma cells [38]. That 1,4-BQ inhibits cell proliferation has been confirmed in several studies [39,40]. In our study, we used MTT to investigate the cell viability and EdU to test the cell proliferation change after 1,4-BQ exposure in both PTP4A3-suppressed cells and control cells. We found that cell viability and DNA replication were more severely suppressed in PTP4A3-inhibited cells compared to normal cells after exposure to 1,4-BQ. This indicates that PTP4A3 was related to the inhibition of cell proliferation caused by 1,4-BQ. The results agree with previous reports that PTP4A3 was involved in cell proliferation [28,29]. 

DNA replication is the vital step of cell proliferation. To investigate whether more DNA damage caused the reduction in DNA replication, we used a comet assay to detect the DNA damage after 1,4-BQ exposure. 1,4-BQ increased the DNA damage in both K562-PTP4A3^−^ cells and K562-NC cells in a dose-dependent manner. 1,4-BQ could cause DNA damage through suppressing type 1 topoisomerases [41]. DNA damage was higher in K562-PTP4A3^−^ cells than K562-NC cells at a low dose 1,4-BQ (10 μM). However, the situation was reversed for the high-dose group (20 μM). PTP4A3 silence promoted DNA damage response without influence of chromosomal stability [42]. Our results showed a different trend of DNA damage at different concentrations of 1,4-BQ, hence how PTP4A3 influenced the DNA damage in 1,4-BQ toxicity needs more evidence to clarify. 

The increase in oxidative stress is an important factor in benzene toxicity. ROS could regulate HIF-1alpha in various disease processes [43]. Therefore, we detect the ROS level after 1,4-BQ treatment. Oxidative stress was improved in both K562-PTP4A3^−^ cells and K562-NC cells in 20 μM 1,4-BQ group. A gentle growing rate of ROS was observed in K562-PTP4A3^−^ cells, while a sharp increase was seen in K562-NC cells after the treatment of 20 μM 1,4-BQ. Overexpression of PTP4A3 was found to reduce the intercellular ROS level in colorectal cancer cells [28]. Further investigations are needed to explain the relationship between PTP4A3 and ROS.

Oxidative stress is a frequent cause of apoptosis [44]. Benzene and its metabolite could induce apoptosis [16,45]. In our results, explicit increase of apoptosis was observed in K562 cells in 20 μM 1,4-BQ group, which is consistent with previous studies. It is noteworthy that down-regulation of PTP4A3 caused more apoptosis than normal cells after exposure to 20 μM 1,4-BQ. PTP4A3 depletion caused apoptosis elevated in triple-negative breast cancer [46]. Suppression of PTP4A3 caused apoptosis in prostate cancer cells and gastric cancer cells as well [27,47]. This indicates that the inhibition of PTP4A3 may be a cause of benzene-induced apoptosis exacerbation. 

Chronic benzene poisoning may cause AML. The survival of AML cells depended on activation of the PI3K/AKT pathway [48]. Previous studies reported that PTP4A3 could activate the PI3K/AKT pathway [49,50]. In addition, the inactivation of PI3K/AKT pathway may induce cell proliferation prohibition and apoptosis acceleration [51,52]. We assumed that PI3K/AKT pathway was inhibited after exposed to 1,4-BQ. As a result, PI3K/AKT pathway was significantly suppressed in both PTP4A3 down-regulated cells and normal cells after 1,4-BQ exposure. This data suggested the suppression of PTP4A3 in K562 cells inhibited PI3K/AKT pathway leading to limited proliferation and elevated apoptosis after 1,4-BQ exposure.

## 5. Conclusions

In summary, HIF-1alpha may regulate PTP4A3 to be involved in benzene hematopoietic poisoning progression. The inhibition of PTP4A3 may aggravate the inhibition of cell proliferation and increase in apoptosis induced by 1,4-BQ through PI3K/AKT signaling pathway. Additional research is required to further explore the exact function and mechanism of PTP4A3 in benzene toxicity.

## Figures and Tables

**Table 1:** Primers used in the article.

Species	Genes	Primers(5′-3′)
Mouse	β-actin	F:CTATGCTCTCCCTCACGCCA
		R: TCACGCACGATTTCCCTCTC
Mouse	PTP4A3	F: CCTGTAAGGCAGCCCCAACTA
		R: GTGTCTTAGCCAGGGTTTTATG
Human	β-actin	F: ATCCGCAAAGACCTGT
		R: GGGTGTAACGCAACTAAG
Human	PTP4A3	F: CAGCCAGTCTTCCACTACCTT
		R: GCTTCCTCATCACCCACAACC
